# The Diversity of Morphological Traits and Seed Metabolomic Composition in Buckwheat Genetic Resources

**DOI:** 10.3390/plants14060903

**Published:** 2025-03-13

**Authors:** Petra Hlásná Čepková, Dagmar Janovská, Maria Bernhart, Pavel Svoboda, Michal Jágr, Vladimir Meglič

**Affiliations:** 1 Gene Bank, Czech Agrifood Research Center (former Crop Research Institute), Drnovská 507/73, Prague 6—Ruzyně, 161 06 Prague, Czech Republic; dagmar.janovska@carc.cz; 2 Saatzucht Gleisdorf GmbH, Am Tieberhof 33, 8200 Gleisdorf, Austria; maria.bernhart@saatzuchtgleisdorf.at; 3 Molecular Genetics, Czech Agrifood Research Center (former Crop Research Institute), Drnovská 507/73, Prague 6—Ruzyně, 161 06 Prague, Czech Republic; pavel.svoboda@carc.cz; 4 Quality of Plant Products, Czech Agrifood Research Center (former Crop Research Institute), Drnovská Prague 6—Ruzyně, 161 06 Prague, Czech Republic; jagr@vurv.cz; 5 Agricultural Institute of Slovenia, Hacquetova ulice 17, SI-1000 Ljubljana, Slovenia; vladimir.meglic@kis.si

**Keywords:** common buckwheat, genetic resources, environment, metabolomic profiling, minor cereal

## Abstract

This study examines the impact of environmental conditions on the growth, yield, and biochemical composition of common buckwheat (*Fagopyrum esculentum* Moench.) across two locations in Central Europe over three consecutive growing seasons (2019–2021). Significant variations in meteorological conditions, including temperature fluctuations and rainfall, were observed between two locations: Austria (AT) and the Czech Republic (CZ). The study highlights the role of these environmental factors in influencing morphological traits such as plant height, leaf dimensions, and 1000-seed weight (TSW), as well as nutritional and bioactive compound content. Buckwheat plants in Austria generally exhibited higher mean values for plant height and TSW compared to the Czech Republic, with significant variability observed across varieties and years. In terms of nutritional quality, crude protein content ranged between 12.56 and 14.71% dw, with the highest protein levels linked to cooler, low-rainfall conditions. The study also investigated phenolic compounds, particularly rutin, which showed a significant increase in content in 2021, likely due to extreme weather conditions. Varieties such as Sweden-1, Tempest, and Zamira exhibited stable, high rutin levels across all years. Overall, this research highlights the complexity of environmental influences on the agronomic and nutritional traits of buckwheat and provides valuable insights for future breeding programs aimed at improving yield and nutritional value under changing climatic conditions.

## 1. Introduction

Buckwheat (*Fagopyrum esculentum* Moench.) is an example of an underutilized crop species despite its abundance of nutritional and bioactive components [1]. With a growing interest in healthy lifestyles, buckwheat is receiving global attention as a component of functional foods. Buckwheat is a rich source of carbohydrates, proteins, fiber, nutrients, and bioactive phytochemicals. The presence of bioactive compounds makes buckwheat an important part of the human diet [2]. Many scientists have highlighted its multifaceted potential as a super crop to address the challenges of food and nutritional security [3,4,5,6]. Due to its nutritional and medicinal properties, the demand for rutin and other flavonoids from buckwheat is increasing in the food, pharmaceutical, and cosmetic industries [7]. However, its cultivation remains limited due to several unfavorable traits, including an indeterminate growth habit, unstable yields, a high rate of flower abortion, poor palatability, and the short shelf life of grains. This necessitates a systematic analysis of the available genetic diversity for economically important traits through the development of core collections and their multi-locational evaluation [4]. The variability of common buckwheat germplasm has been confirmed in previous studies [8,9]. Moreover, recent scientific studies [10,11,12] have focused on evaluating morphological and phenological traits and assessing the genetic diversity of buckwheat genetic resources from different regions. Buckwheat accessions cultivated in pot experiments in Slovenia exhibited significant agro-morphological variation, likely influenced by environmental factors [10]. Similarly, buckwheat landraces from various provinces in Korea displayed morphological variability at each location [13]. Additionally, significant variability in morphological traits was observed across locations in the northwestern Himalayas [14]. Conversely, the genetic variance of Korean common buckwheat germplasm was found to be narrow [12].

Moreover, plant breeders face multiple global challenges that affect food security, productivity, accessibility, and nutritional quality. One major challenge is development of environmentally resilient crop cultivars in response to rapid shifts in cultivation conditions and resource availability caused by climate change [15,16].

Plants produce a variety of metabolites that are essential for plant growth and human health. The comprehensive and comparative identification of metabolites across different plant varieties can improve our understanding of metabolite production in other species and help identify the specific metabolites with health-promoting functions from the specific plants [17]. Recently, three key metabolomic studies [17,18,19] utilizing targeted metabolomic profiling in buckwheat were published. In the first study, metabolite differences between red- and white-flowered buckwheat were analyzed [18]. In the second one, 722 metabolites were identified in the seeds of four common and four Tartary buckwheat varieties from China [17], revealing differences between the two buckwheat species. In third study a unique set of 136 common buckwheat accessions was cultivated and evaluated under the conditions of Central European conditions [19].

Understanding the vast variability in the nutritional components of buckwheat germplasm cultivated under diverse global conditions highlights the importance of conserving and safeguarding this genetic wealth. It also emphasizes the need to select exceptional accessions suited to various environments, providing the potential to develop new buckwheat varieties adapted to different climates and production systems [4].

In this study, we evaluated 25 selected agro-morphological, chemical, and metabolic traits of 53 common buckwheat genotypes cultivated in two locations in Central Europe (Czech Republic and Austria) over three consecutive growing periods (2019–2021). We hypothesized that variations in weather conditions significantly impact the agro-morphological traits of buckwheat genotypes and that their chemical and metabolic profiles in the seeds vary, reflecting underlying genetic diversity across different environments.

## 2. Results and Discussion

### 2.1. Morphological and Phenological Evaluation

Buckwheat germplasm has been characterized globally for various qualitative and quantitative traits using plant descriptors developed by the International Plant Genetic Resources Institute (IPGRI), Biodiversity International, and others [20]. Our study evaluated selected morphological and phenological traits of buckwheat collections and obtained unique data on the growth and development of 53 buckwheat accessions under different environmental conditions. Due to the lower yield potential of common buckwheat compared to major crops, new breeding goals for its improvement include increasing pre-harvest sprouting resistance, reducing shattering losses, introducing self-incompatibility, and developing ecotypes and semi-dwarf varieties [21].

**Figure 1:**
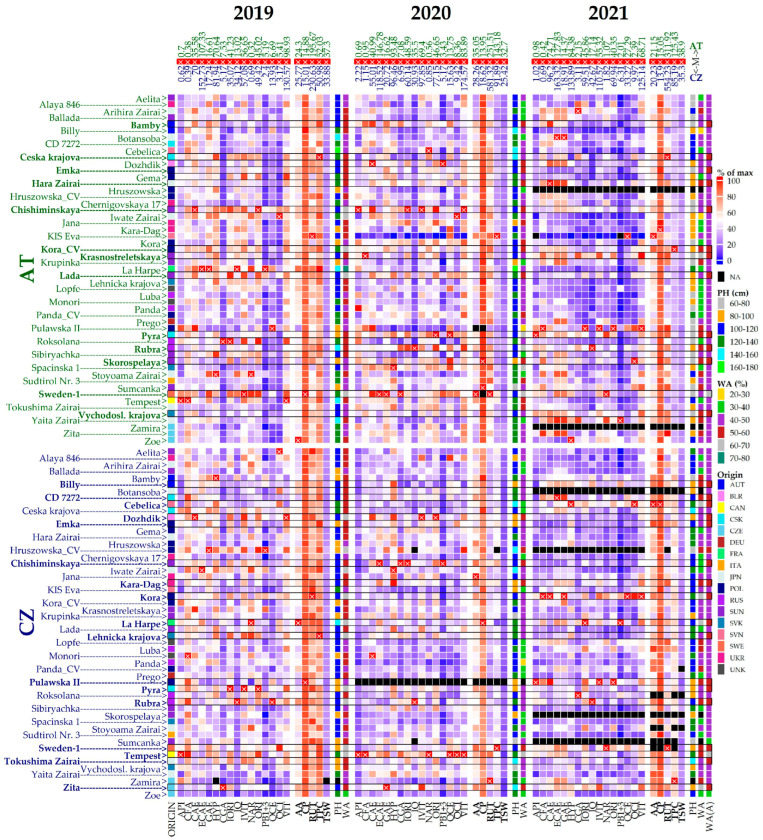
Buckwheat accessions are listed on the left side of the plot. Mentioned descriptors comprise plant height (PH [cm]), antioxidant activity (AA [µmol TE/g dw), thousand seeds weight (TSW [g]), crude protein (CP [% dw]) and content of total polyphenols (TPC [g GAE/kg dw]) and selected fractions, namely apigenin (API), caffeic acid (CFA), isoquercetin (IQ), hyperoside (HYP), gallic acid (GAE), catechin (CAE), procyanidins B1 + B3 (PB1 + 3), chlorogenic acid (CGA), epicatechin (ECAE), orientin (ORI), isoorientin (IORI), vitexin (VIT), isovitexin (IVIT), naringenin (NAR), rutin (RUT), quercitrin (QCI), quercetin (QCE), all measured in µg/g dw. Main heatmap bodies correspond to values of 21 descriptors (excluding PH) depicted as percentages of maximal value (listed above the heatmap, marked with ×) recorded for respective descriptor in the given year of observation and displayed on a scale from blue (0%) to red (100%). Side heatmaps contain information about plant heights (PHs) for each accession as well as a score based on the weighted average (WA) of ratios to maximum values in each descriptor, displayed both on a categorical scale. Accessions with the best overall WA mean [WA(A)] are highlighted within the heatmap. Origin stands for the country of origin of respective accession.

Various traits, including plant height, number of branches, number of flowers, 1000-seed weight, plant type, stem color, flower color, seed color, and seed shape, along with essential quality characteristics, are of paramount importance in buckwheat breeding. Globally, a broad range of variability has been observed in germplasm for different yield-contributing traits [20]. Morphological and phenological data collected over three years at both locations are summarized in Tables S1 and S2 and the variability of the observed traits is presented in Figure 1.

**Figure 2:**
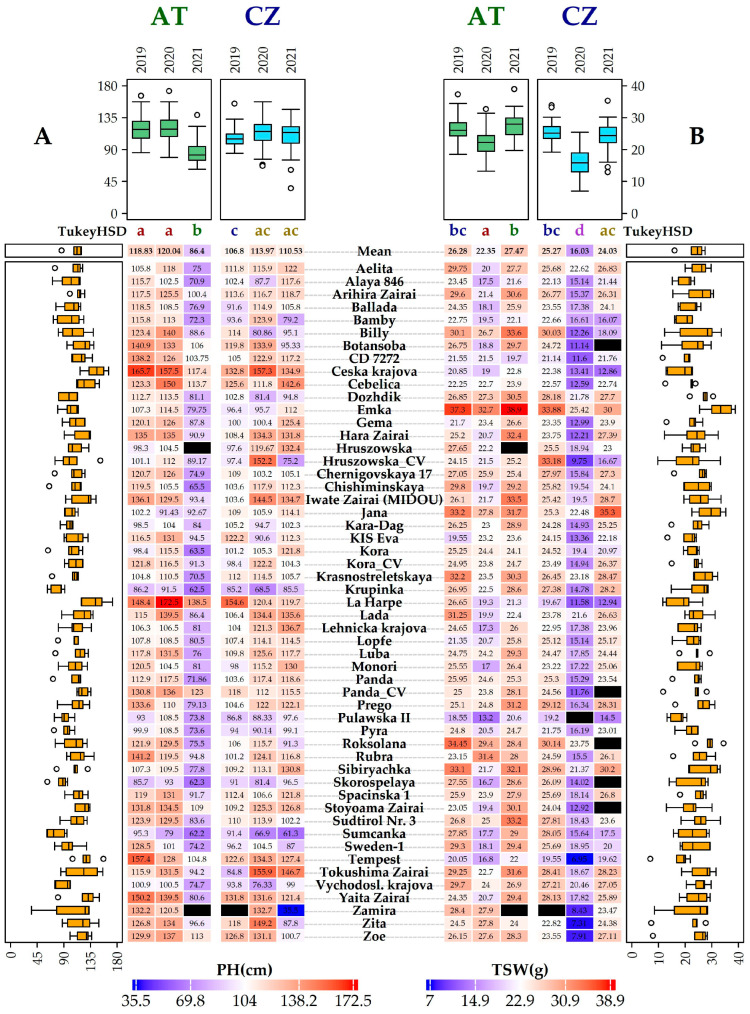
Heatmap (**A**) shows the plant height (PH, cm) and heatmap (**B**) shows thousand seed weight (TSW, g) for the accession in the respective year. Values for respective traits are displayed on a scale from blue (min) to red (max) for each accession in the respective year. Years with significantly different means are depicted by different letters (Tukey’s HSD) above each heatmap. Boxplots above and next to each heatmap display the distribution of values across individual years and accessions, respectively. Black rectangles indicate the missing values for the given trait in the given accession.

One of the evaluated morphological traits in this study was plant height (PH) (Figure 2). The highest mean plant height was recorded in both locations in 2020, with values of 114.01 ± 12.00 cm in Prague (CZ) and 120.04 ± 16.81 cm in Gleisdorf (AT). The lowest mean PH for the tested buckwheat collection in both locations was 104.80 ± 12.22 cm, observed in the Czech Republic in 2019 and in Austria in 2021. This value is very close to the mean value of PH observed in buckwheat accessions cultivated in Slovenia [10]. The mean plant height of 964 Chinese common buckwheat materials was 107.40 cm [9]. Significant variability in PH was observed across the assessed buckwheat samples. The highest plant height was recorded in the variety La Harpe (2020; AT) at 172.50 ± 16.47 cm in 2020 in AT, while the variety Zamira (2019; CZ) exhibited the shortest height of 35.50 ± 9.95 cm in 2019 in CZ. In 2019 and 2020, the mean PH values were higher in AT, whereas in 2021, they were higher in CZ. In 2019, CZ experienced the highest temperatures among the tested years, accompanied by excessive rainfall. Conversely, locality AT experienced extreme rainfall from June to August 2021. The mean crop height (CRH) ranged from 74.34 cm to 84.92 cm in CZ over three years, while in Austria, exhibited greater variation, ranging from 50.83 cm to 90.09 cm. Leaf dimensions remained consistent across years and locations, with leaf blade length and width ranging from 5.00 to 6.00 cm. Extreme values were measured in AT in 2020, with leaves reaching around 8 cm in width and length. In contrast, measurements from AT in 2021 indicated much smaller leaves, with a mean leaf blade length and width of 2.70 cm and 2.33 cm, respectively. Ghiselli et al. [22] observed significant variability in morphological traits among buckwheat varieties in response to different environmental conditions.

**Figure 3:**
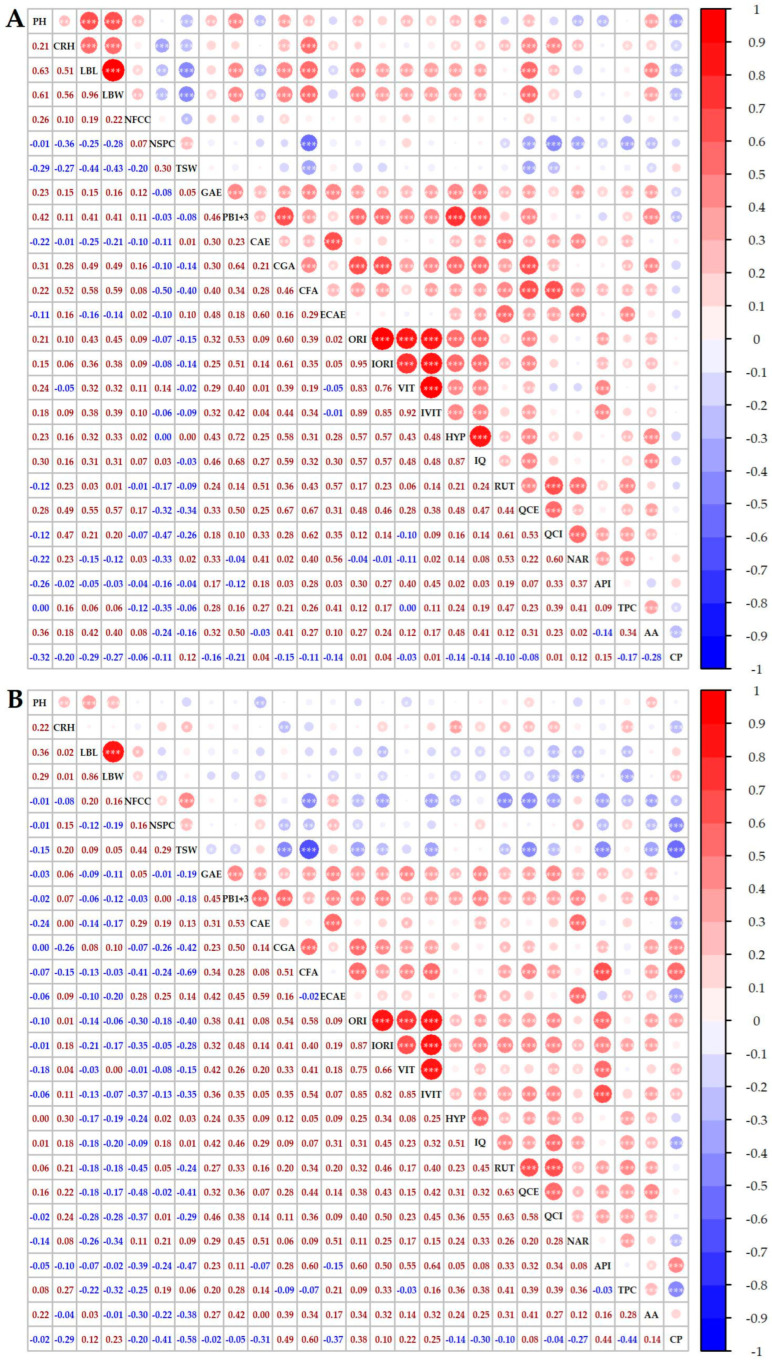
Spearman’s correlation among evaluated descriptors for the collection of selected buckwheat genotypes cultivated in Austria (**A**) and the Czech Republic (**B**). The colors of the circles above the diagonal indicate whether the correlation between the pair of descriptors is negative (blue)/positive (red), while their magnitude is proportional to Spearman’s ρ indicated below the diagonal. Significant correlations are represented by * (*p* < 0.05), ** (*p* < 0.01), and *** (*p* < 0.001), respectively.

Throughout the study period, the mean values of 1000-seed weight (TSW) were consistently higher at the Austrian location (Figure 2). Specifically, the mean TSW values in Austria were 26.25 g, 22.35 g, and 27.29 g from 2019 to 2021. In CZ, the mean TSW values were comparatively lower, measuring 24.64 g, 16.00 g, and 24.03 g during the same years. However, these values are similar to those observed in the Slovenian samples [10]. These mean values exceeded those reported for 322 buckwheat accessions in India, where TSW averaged 20.56 g [7]. Notably, a TSW greater than 25 g has been identified as a valuable trait in buckwheat germplasm [20]. Statistical analyses revealed significant differences in TSW both between the two locations and among varieties, consistent with findings from the mountainous region of Italy [22] and various buckwheat varieties in Northern England [23]. The year 2020 was less favorable for achene formation in both locations, with the lowest TSW value recorded in the Canadian variety Tempest (5.59 g) in CZ, while the highest value was observed in Emka (38.9 g) in AT in 2021. The observed range of TSW was notably broader than the previously reported range (21.00–42.00 g) assessed in 251 buckwheat accessions [8]. Additionally, a significant negative correlation between TSW and the number of days to flowering, as reported by Rauf et al. [8], was mirrored in this study. Furthermore, a robust negative correlation was confirmed between TSW, CFA, and QCE (Figure 3B).

Mentioned descriptors comprise plant height (PH), crop height (CRH), leaf blade length (LBL), leaf blade width (LBW), number of flowers clusters per cyme (NFCC), number of seeds per cyme (NSPC), 1000-seed weight (TSW), gallic acid (GAE), procyanidine b1+b3 (PB1+3), catechin (CAE), chlorogenic acid (CGA), caffeic acid (CFA), epicatechin (ECAE), orientin (ORI), isoorientin (IORI), vitexin (VIT), isovitexin (IVIT), hyperoside (HYP), isoquercetin (IQ), rutin (RUT), quercetin (QCE), quercitrin (QCI), naringenin (NAR), apigenin (API), total phenolic content (TPC), antiox. activity (AA) and crude protein (CP).

### 2.2. Chemical Compounds Analysis

**Figure 4:**
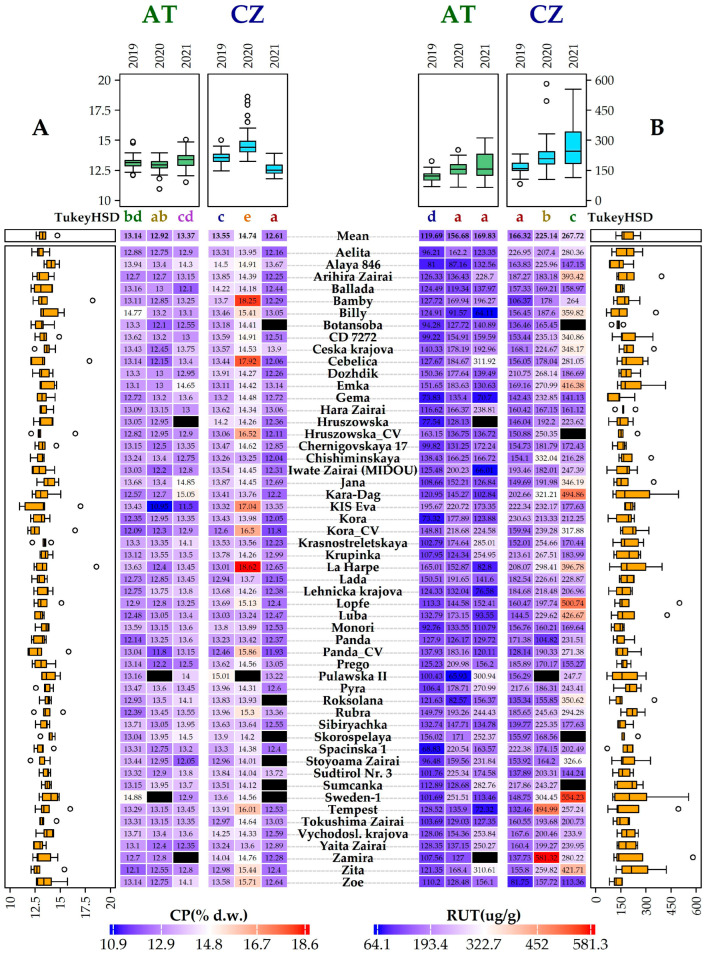
Heatmap (**A**) shows the protein content (PC, % dw), while heatmap (**B**) shows the rutin content (RUT, µg/g dw) for the accession in the respective year. Values for respective traits are displayed on a scale from blue (min) to red (max) for each accession in the respective year. Years with significantly different means are depicted by different letters (Turkey’s HSD) above each heatmap. Boxplots above and next to each heatmap display the distribution of values across individual years and accessions, respectively. Black rectangles mean the missing values for the given trait in the given accession.

The crude protein (CP) content in buckwheat ranged between 5.70 and 14.10 g/100 g of fresh weight such as quinoa and amaranth, while exceeding values observed in cereal grains [4]. In common buckwheat germplasm, seed protein levels above 12.2 mg/100 mg are considered promising [3]. The range of CP was similar in both locations, varying from 12.91 ± 0.18% dw (2020) to 13.36 ± 0.29% dw (2021) in AT and from 12.56 ± 0.22% dw (2021) to 14.71 ± 0.20% dw (2020) in CZ (Figure 4).

The protein content depends on the variety, with an extreme value of around 18% [24]. In this study, significant differences in CP content were observed between buckwheat genotypes, growing seasons, and locations. These findings partially align with the results of a study conducted in Poland [25], which found significant differences in protein content between growing seasons and localities but not among the four studied buckwheat genotypes. The high CP content in CZ in 2020 resulted from a combination of low temperatures after sowing, slow initial growth, and very low rainfall in July 2020 which corresponds to the findings of Gavrić [26]. The higher protein content can also be attributed to an extended period of temperatures exceeding 22 °C [27]. Water availability is particularly crucial in the last days of vegetation, as it directly influences the final protein content in the formed seeds [28,29]. Conversely, the lowest mean CP value (12.61 ± 0.22% dw) in CZ in 2021 and (12.91 ± 0.18% dw) in AT in 2020 may have been influenced by heavy rainfall at the beginning of the vegetative period (May), which could have weakened the plants’ initial growth. The negative correlation was confirmed between CP and TSW (Figure 3). In CZ in 2020, a remarkably high mean CP was recorded while TSW value was very low, which was consistent with the observed negative correlation between CP and TSW, TPC, and NSPC. On the other hand, a stronger positive correlation was observed between CP and API, CGA, and CF.

Considering the buckwheat varieties and two locations, the total phenolic content did not exhibit significant variation. However, statistically significant differences among locations, years, and varieties were confirmed, similar to findings in buckwheat grains cultivated in northeastern Slovenia [30]. The mean values for all years and both locations ranged from 3.93 mg GAE/g dw (CZ, 2019) to 4.98 mg GAE/g dw (CZ, 2021), with the highest values attributed to KIS Eva, Sweden-1, Tempest (AT, 2020), Kora_CV (AT, 2021), Tempest (CZ, 2020), and Zamira (CZ, 2021). The values of TPC for AT (2.78, 3.82, and 3.98 mg GAE/g dw) and CZ (3.05, 3.41, and 3.83 mg GAE/g dw) along with AA values for AT (13.14, 15.96, and 13.75 umol TE/g dw) and CZ (12.85, 16.89, and 11.91 umol TE/g dw) were remarkably similar to those reported by Terpinc et al. [31] in raw buckwheat seeds. The highest mean AA values were found in 2020, where the varieties Sweden-1 and Tempest performed well in both locations, with AA values close to or exceeding 20 umol TE/g dw. A positive correlation was observed between TPC and AA. However, the correlation between TPC and AA with the monitored phenolic compounds is positive but very weak (Figure 3), confirming that the major phenolic compounds contribute only partially to the overall AA [32].

### 2.3. Metabolomic Profiling in Buckwheat Accessions

Metabolomics is one of the ‘omics’ approaches that have been extensively applied to crop improvement, as metabolites are unique to plants and play an important role in crop yield and nutritional quality [33]. This study used metabolomics as a powerful tool to evaluate the phenotypic, morphological, and nutritive composition within buckwheat samples grown under different environmental conditions. Plants produce the enormous secondary metabolites and signaling molecules in response to stress or defensive signaling. These metabolites vary across genotypes, taxonomic groups, and physiology, and the experimental technologies used to assess them differ [34]. Drought and temperature stresses are abiotic factors that significantly affect plant growth and development, causing numerous changes in the physiology and biochemistry of plants [35], including increased production of various classes of secondary metabolites [36]. It has been previously confirmed that external factors significantly influence secondary metabolism processes, particularly flavonoid biosynthesis, in buckwheat plant tissue [37]. The synthesis of flavonoid compounds is complex, and their metabolic pathways are regulated by a series of key enzymes. Plant flavonoids play crucial roles in numerous biological processes, including protection against biotic agents (such as viruses, fungi, and bacteria), functioning as chemical messengers in symbiosis with mycorrhiza and bacteria, contributing to flower pigmentation to attract pollinators, and exhibiting allelopathic properties [38].

In our study, rutin, epicatechin, hyperoside, vitexin, isovitexin, and catechin were identified as the dominant phenolic compounds found in buckwheat seeds. Orientin, isoquercetin, quercetin, and gallic acid were present in smaller amounts compared to the more abundant compounds. Trace amounts of chlorogenic acid, hesperidin, naringenin, and apigenin were also detected. Rutin (quercetin-3-rutinoside) was verified as a dominant phenolic compound not only in grains but also in other plant parts [37,39] representing 90.4% of total flavonol content [40]. Previous reports have indicated a significant variability in rutin content across different buckwheat varieties [18,41,42]. Moreover, improving rutin content has been an objective of buckwheat breeding for several years [43].

The mean rutin content ranged from 119.87 ± 2.66 µg/g dw (AT, 2019) to 276.57 ± 6.53 µg/g dw (CZ, 2021). The highest mean rutin content was reached in 2021 at both locations (Figure 4). The rutin content in samples from these two European localities was comparable to that of varieties grown in Poland [41], Ukraine [44] and Japan [43] or even higher than the values reported in previous studies [31,45] contrary to the very low levels observed in commercial buckwheat samples from Turkey [46]. The varieties Sweden 1 (554.23 ± 4.59 µg/g dw), Zamira (581.32 ± 7.42 µg/g dw), and Tempest (499.95 ± 10.66 µg/g dw) exhibited the highest rutin content in 2021, whereas Zoe reached 81.75 ± 5.23 µg/g dw in 2019 in CZ. In AT, the variety Pulawska II (300.94 ± 12.62 µg/g dw) and Zita (310.31 ± 29.33 µg/g dw) showed the highest rutin content, whereas Billy exhibited significantly lower levels (64.11 ± 0.54 µg/g dw) in 2021. The rutin content was positively correlated with quercetin and quercitrin but negatively correlated with the number of flower clusters per cyme and TSW (Figure 3). In general, rutin levels were higher in buckwheat samples from CZ than from AT. This may be due to higher summer temperatures and lower rainfall in CZ, which could have caused greater plant stress. Drought often leads to oxidative stress, resulting in formation of reactive oxygen species (ROS). To counteract this, plants increase the synthesis of antioxidants like rutin, which help neutralize ROS and protect plant cells from damage [47].

There are contrasting reports regarding the accumulation or reduction of flavonoid compounds in plants under light and temperature stresses [48]. In both European localities, the highest rutin values were recorded in 2021, probably due to more extreme weather conditions, where the dry season was followed by heavy rainfall (CZ: May 2021, AT: June-August 2021), increasing plant stress. Stable and high rutin contents were observed in the varieties Sweden-1, Tempest, Zamira, Kara-Dag, and Emka (Figure 4) across all three years in CZ.

Certain environmental factors, such as higher altitude and light irradiation, may contribute to increased rutin content and other phenolic compounds [5,49,50]. However, this theory cannot be confirmed in this study, as the altitude difference between the two locations was only 24 m. Although AT is located further south, higher level of most phenolic compounds was observed in CZ.

Hyperoside, a flavonol glycoside, along with epicatechin and vitexin from the flavon group, was identified as one of the most abundant phenolic compounds in buckwheat seeds, similar to those found in buckwheat samples grown in southern Italy [51]. Their detected levels in buckwheat seeds were below 160 µg/g dw. Higher hyperoside content was observed in locality CZ over the three years, with the highest mean value (60.07 ± 2.22 µg/g dw) recorded in 2021. According to the results, variety Špačinská 1 in AT (93.48 ± 1.52 µg/g dw) and Iwate Zairai in CZ (96.46 ± 1.05 µg/g dw) had the highest hyperoside levels in 2020. The hyperoside content showed a strong correlation with isoquercetin and procyanidins B1 + B3. Along with other compounds in buckwheat, hyperoside contributes to its antioxidant properties and may offer several health benefits, such as supporting cardiovascular health and providing anti-inflammatory effects [52].

The mean levels of epicatechin remained relatively stable across different years and locations, ranging from 41.72 ± 0.57 µg/g dw (AT, 2019) to 69.70 ± 1.31 µg/g dw (CZ, 2021), which corresponds to the previously published epicatechin content [5]. However, individual accessions could reach more than double these values. The highest epicatechin levels were recorded in the varieties Zita (121.91 ± 1.32 µg/g dw) in AT in 2021 and CD 7272 (163.32 ± 3.58 µg/g dw) in CZ in 2021. In previous studies, its presence was confirmed in buckwheat seed and seed husk [37].

The mean vitexin content in 53 accessions varied between 35.59 ± 1.27 µg/g dw (AT, 2021) and 71.96 ± 1.12 µg/g dw (CZ, 2020). The highest vitexin content was observed for the varieties Tempest (174.57 ± 3.26 µg/g dw) in CZ in 2021 and Gema (94.33 ± 1.19 µg/g dw) in AT in 2019. The vitexin levels in seeds from both locations closely resembled those found in the flowers of Tartary buckwheat [53]. Orientin, isoorientin, and isovitexin were identified in different parts of eight common buckwheat genotypes in similar amounts [54] and are the major phenolic compounds induced by malting [31]. Catechin levels were very similar to those of orientin and isoorientin, with slightly higher mean values observed in CZ over the years. A strong positive correlation was confirmed between orientin, isoorientin, vitexin, and isovitexin. Gallic, chlorogenic, and caffeic acids were found in trace amounts. Gallic acid levels ranged from 7.39 ± 0.82 µg/g dw (AT,_2021) to 9.13 ± 0.21 µg/g dw (AT, 2019), chlorogenic acid from 2.17 ± 0.54 µg/g dw (AT,2020) to 0.74 ± 0.02 µg/g dw (AT,_2021), caffeic acid between 0.48 ± 0.01 µg/g dw (AT, _2020) and 0.21 ± 0.01 µg/g dw (AT, 2019). The mean values of these three phenolic acids detected in samples from CZ over the years fell within the previously mentioned ranges.

In the studied genotypes, the significant variability in the accumulation of evaluated secondary metabolites across years and different environments was confirmed. Findings from a recently published study indicate that drought triggers a complex regulatory network involving genes and metabolites in buckwheat, leading to significant alterations in both primary and secondary metabolites [47]. This suggests that buckwheat undergoes a coordinated metabolic adjustment to cope with drought stress, potentially enhancing its resilience through the accumulation of specific metabolites that play protective roles. Moreover, the biosynthesis and accumulation of bioflavonoids highlight their essential role in plants, with their structural and quantitative composition varying depending on the species and variety of the crop [37].

## 3. Materials and Methods

### 3.1. Plant Material and Weather Conditions

Fifty-three common buckwheat accessions were used in this study (Table S3). 41 common buckwheat accessions were provided by gene banks, nine were from commercial varieties, three were from the Czech Gene Bank working collection and four were control varieties. In the years 2019, 2020, and 2021, all accessions were grown in two localities of Central Europe—Prague, Czech Republic (CZ) and Gleisdorf, Austria (AT). Prague is situated in Central Bohemia, the experimental fields are located on the northwest side of Prague (50°05′11.4″ N, 14°18′11.3″ E, and 338 m. a. s. l.) with an elevation of 320 m, annual precipitation of 472 mm, and mean annual air temperature of 8.4 °C. The soil is Chernozem (IUUS/ISTRIC/FAO, 2006) with a loamy clay texture. The precrop was winter wheat fertilized with 100 N kg ha-1 (UREALstabil, AGRA GROUP Inc., Střelské Hoštice, Czech Republic) before sowing. Gleisdorf is a town of the Austrian province Styria (47°06′53.6″ N, 15°42′29.3″ E, and 362 m. a. s. l.). Gleisdorf is characterized by an elevation of 368 m, an annual precipitation of 797 mm and a mean annual air temperature of 8.9 °C. The soil is alluvial soil with a loamy silt texture. The precrops in 2019, 2020, and 2021 were corn and soybean, respectively. At both sites the artificial herbicides and pesticides were not applied. In both localities, buckwheat samples were sown in two rows of 1 m length, 25 cm apart, with 50 seeds per row. Original seeds were used for sowing in all three years at both sites, and plot isolation was not used. During the growing season, selected morphological and phenological traits were evaluated according to the descriptors for buckwheat (*Fagopyrum* spp.) [55]. A representative sample of 10 g was taken from the harvest and delivered to the laboratory. The buckwheat seeds were stored in a dark and freezing place (−18 °C) before prior to further processing.

**Figure 5:**
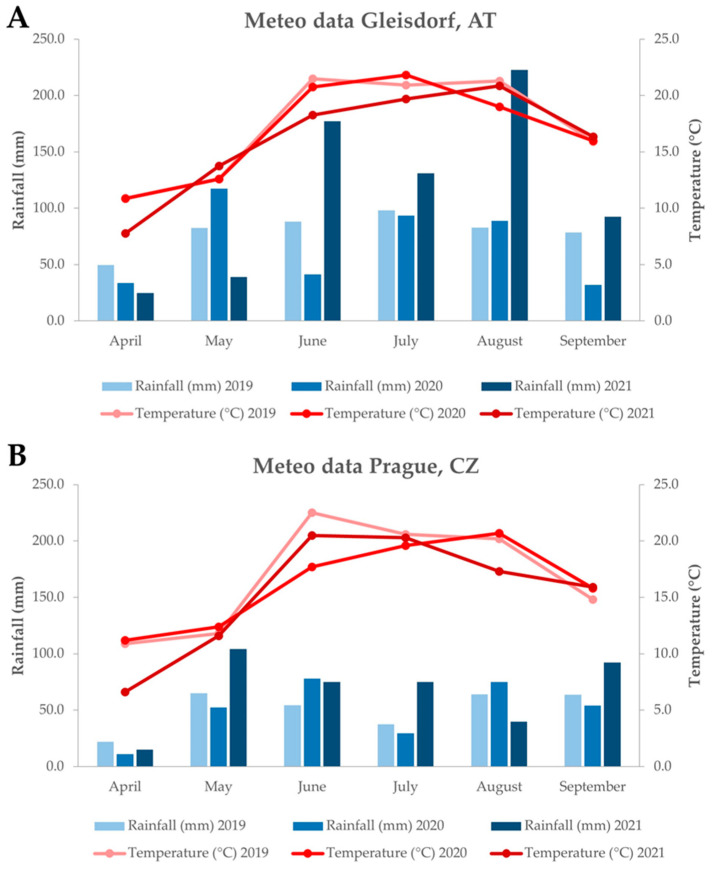
Weather conditions in Gleisdorf, Austria (AT) (**A**) and Prague, Czech Republic (CZ) (**B**) in 2019–2021.

Meteorological data (Figure 5) from the growing seasons at both sites showed differences in the total precipitation and temperature across all years and locations. In AT, the cumulative precipitation during the growing seasons (from April to September) was significantly higher compared to CZ. The most notable variations in rainfall between the localities occurred in July 2019 (the difference was 61 mm), July 2020 (64 mm), June 2021 (102 mm), and August 2021 (183 mm). Fluctuations in mean temperatures throughout the growing period were generally minimal. However, unlike the other seasons, the lowest temperatures were observed in CZ in 2020, whereas AT experienced the highest temperatures in 2021.

### 3.2. Chemicals

Standards of the phenolic compounds apigenin, caffeic acid, catechin, chlorogenic acid, epicatechin, gallic acid, hesperidin, hyperoside, isoorientin, isoquercetin, isovitexin, naringenin, orientin, procyanidins (B1 + B3), quercetin, quercitrin, rutin, vitexin, and the internal standard probenecid were purchased from Sigma-Aldrich (St. Louis, MO, USA). Methanol (LC-MS grade, ≥99.9%) was obtained from Riedel de Haën (Seelze, Germany). Formic acid (LC-MS grade, 99%) was purchased from VWR (Leuven, Belgium). Pure water was obtained from a Milli-Q purification system (Millipore, Bedford, MA, USA).

### 3.3. Standards Preparation and Sample Isolation

To prepare reference stock solutions, individual reference standards of phenolic compounds were dissolved in MeOH to obtain a stock solution of 0.5 mg/mL and stored at −18 °C. The stocks were further diluted with methanol in a concentration range of 0.001–2.000 µg/mL to create calibration curves for phenolic compound quantification. Probenecid was dissolved in MeOH at 0.5 mg/mL to prepare a stock solution of the internal standard. Probenecid was then added to individual reference standard solutions or test samples to achieve a final concentration of 0.1 µg/mL.

Buckwheat grains were pre-milled with an IKA A11 basic mill (IKA-Werke, Staufen, Germany). They were then frozen in liquid nitrogen and ground using a mortar and pestle until a fine powder was obtained. The powder was placed in sealed plastic bags and stored at −18 °C. The extraction procedure of phenolic compounds was based on a procedure modified according to Qin et al. [42]. The extracts were then stored at −18 °C prior to UHPLC-ESI-MS/MS analysis.

### 3.4. UHPLC-ESI-MS/MS Instrumentation

The chromatographic system (Dionex UltiMate 3000 UHPLC system, Dionex Softron GmbH, Bayern, Germany) consisted of a binary pump (HPG-3400RS), an autosampler (WPS-3000RS), a degasser (SRD-3400), and a column oven (TCC-3000RS). Detection was performed on a quadrupole/orbital ion trap Q-Exactive mass spectrometer (Thermo Fisher Scientific, San Jose, CA, USA). Analytes were separated on a reversed phase Acentis Express C18 column (2.1 × 100 mm, 2.7 µm) from Supelco (Bellefonte, PA, USA). The LC-MS system was equipped with a heated electrospray ionization source (HESI-II) and Xcalibur software, version 4.0.

### 3.5. UHPLC-ESI-MS/MS Analysis

Chromatographic separation was performed as described by Janovská et al. [19]. Briefly, gradient elution with 0.2% formic acid in water as solvent A, and methanol with 0.2% formic acid as solvent B was used. The separation was started by running the system with 99% of solvent A + 1% of solvent B; followed by gradient elution to 40% A + 60% B at 11 min. The column was then eluted for 2 min with 100% of B. Equilibration before the next run was achieved by washing the column with 99% A + 1% B for 2 min. The total analysis time was 15 min. The column was maintained at 40 °C, with a flow rate of 0.35 mL/min and an injection volume of 1 µL.

Ionization was performed in the negative electrospray ionization (ESI) mode. The spray voltage was maintained at −2.5 kV. The sheath gas flow was set to 49 arbitrary units, the auxiliary gas flow rate was kept at 12 arbitrary units, and the sweep gas flow was two arbitrary units. The capillary temperature was 260 °C. Nitrogen was used as the sheath, auxiliary, and sweep gas. The heater temperature was kept at 419 °C, and S-lens RF level was 30. The mass spectrometer was generally operated in parallel reaction monitoring (PRM) mode. The precursor ions in the inclusion list were isolated within a retention time window ± 60 s, filtered in the quadrupole at the isolation window (target *m*/*z* ± 0.8 *m*/*z*), and fragmented in an HCD collision cell. Product ions were collected in the C-trap at a resolution 17,500 FWHM, an AGC target value of 1 × 10^6^, and a maximum injection time of 50 ms. The normalized collision energy (NCE) was optimized for each compound. The monitored precursor and daughter ions, retention times, and NCE values are shown in Table S4. The accuracy and calibration of the Q-Exactive Orbitrap LC-MS/MS were checked using a reference standard mixture obtained from Thermo Fisher Scientific. Data were evaluated by the Quan/Qual Browser Xcalibur software, v 4.0.

### 3.6. Determination of Phenolic Compound Concentration in Buckwheat Samples and Statistical Analysis

The identification of phenolic compounds in buckwheat samples was based on their retention times relative to the authentic standards and on mass spectral data (accurate mass determination generating elemental composition and fragmentation patterns of a molecular ion) obtained by LC-MS, which were compared with those described in previous studies conducted on Orbitrap analysis of phenolic compounds [5,19,43].

Calibration curves were constructed by plotting the peak area (adjusted by probenecid as an internal standard) versus the concentration of relevant reference standards. Data analysis was performed in Statistica 12 software (TIBCO Software, Palo Alto, CA, USA).

### 3.7. Chemical Analyses

Kjeldahl analysis: Approximately 10 g of seeds from each tested accession were crushed in a grinding mill (IKA A11 basic, IKA^®^ Werke GMBH and Co., KG, Staufen im Breisgau, Germany) to create individual samples. The dry matter of 5 g of seed samples was further dried in an electric hot air drier at 105 °C for 4 h, according to the standard method AACC International Method 44-11.01. The crude protein content from each sample was determined using the classic Kjeldahl mineralization method and calculated with a conversion factor 6.25 (ICC standard no. 105/2).

Folin Assay: TPC was determined spectrophotometrically with the Folin–Ciocalteu reagent. A modified method of Holasova et al. [56] was used. Two grams of lyophilized sample were extracted with 20 mL of 80% MeOH for 60 min in a centrifuge tube. The tubes were protected from sunlight by aluminum foil. The resulting extract (0.5 mL) was pipetted into a 50 mL volumetric flask and diluted with distilled water. Then, 2.5 mL Folin–Ciocalteu reagent (PENTA, Prague, Czech Republic) and 7.5 mL 20% sodium carbonate solution were added after agitation. After standing in the dark at laboratory temperature for 2 h, absorbance at a wavelength λ = 765 nm was measured against a blank on the spectrophotometer Thermo GENESYS™ 10UV UV-Vis (Thermo Scientific, Waltham, MA, USA). The results were quantified using a gallic acid standard (Merck, Germany) and expressed as gallic acid equivalents (GAE).

DPPH Assay: The radical scavenging capacity (RSC) was determined on microtiter plates using MeOH extracts and the stable radical 2,2-diphenyl-1-picrylhydrazyl (DPPH) [57]. Briefly, 20 mL of MeOH was added to 1 g of sample and shaken for 90 min while it was protected from light by aluminum foil. Twenty µL of the extract reacted for 10 min with 150 µL of DPPH solution with an initial absorbance A = 0.6 at 550 nm. The reaction took place in the dark, and the absorbance at 550 nm was measured afterward using a spectrophotometer (Sunrise absorbance reader, Tecan, Switzerland). The ability to scavenge the DPPH radical was determined using a standard curve obtained with Trolox (Sigma-Aldrich, Hamburg, Germany) in the concentration range from 0.0 to 0.2 mmol/L. The results were expressed as Trolox equivalent (TE) AA (Sigma-Aldrich, Hamburg, Germany).

The analyses were completed in two repetitions for each sample.

### 3.8. Statistical Analyses

Each descriptor was evaluated for a set of 53 buckwheat genotypes and was measured in at least three replicates. Statistical analysis was primarily performed using the R program (R Development Core Team 2020) and Microsoft Office Excel v. 2016. Raw data for each descriptor were analyzed using the Shapiro–Wilk normality test from the “stats” package. A two-way analysis of variance (ANOVA) was applied to the data to test whether year and genotype had a significant effect on individual descriptor scores. To compare each accession in relation to each descriptor, the means and standard deviations for each descriptor were calculated separately for each accession and year of observation. Spearman’s rank correlation was also calculated for each pair of descriptors based on the mean values to test correlations between individual descriptors. To test whether the correlation coefficient was significantly different from zero, the cor.test function was applied. To evaluate individual accessions across all descriptors, a different approach was taken. For each descriptor except PH, the percentage of the maximum value of each descriptor was calculated separately for each accession and year of observation. Based on these percentages, the score for each accession was calculated using the weighted average equation. Each percentage value was multiplied by a coefficient to magnify the value of the primary descriptors. The coefficient for the descriptors was set as follows: 2—QCE, 3—RUT, 4—AA, CP, TPC, TSW, 1—rest of the descriptors.

## 4. Conclusions

Both genetic and environmental factors contribute to variations in secondary metabolite production. The interaction between genetic regulation and environmental cues results in dynamic changes in metabolite profiles. The evaluation of phenotypic and morphological traits in 53 buckwheat accessions showed their variability in response to varying weather conditions. This plasticity enables plants to optimize their defensive and adaptive responses. In the Czech Republic, buckwheat grains exhibited a higher accumulation of phenolic compounds, especially in 2021, with rutin content reaching levels that matched or exceeded previously reported values. In contrast, in Austria, heavy rainfall during the growing season of 2021 negatively impacted plant height and leaf size, with a noticeable reduction in the levels of most evaluated phenolic compounds. Despite these environmental challenges, 15 varieties, including Billy, CD 7272, Cebelica, Dozhdik, Emka, Chishiminskaya, Kara-Dag, La Harpe, Lehnicka krajova, Pulawska II, Pyra, Rubra, Sweden-1, Tempest, Tokushima Zairai, and Zita, demonstrated exceptional performance in the Czech Republic. In Austria, 14 varieties, including Bamby, Ceska krajova, Emka, Hara Zairai, Chishiminskaya, Kora_CV, Krasnostreletskaya, Lada, Pyra, Rubra, Skorospelaya, Sweden-1, and Vychodoslovenska krajova, exhibited strong growth and high scores in the evaluated traits. The observed variability in both morphological and metabolomic traits is crucial for ongoing buckwheat improvement, providing breeders valuable options to develop new varieties and hybrids capable of thriving in diverse environmental conditions. In particular, the selection of buckwheat genotypes with a high flavonoid content, especially rutin, and resistance to stress is important. This will contribute to the continued advancement of buckwheat cultivation and breeding practices.

## Data Availability

The original contributions presented in the study are included in the article/Supplementary Materials, further inquiries can be directed to the corresponding author.

## Supplementary Materials

The following supporting information can be downloaded at: https://www.mdpi.com/article/10.3390/plants14060903/s1, Table S1. Evaluation of selected morphological traits and chemical compounds (expressed as mean value ± sd) in buckwheat seed in Czech Republic in 2019–2021; Table S2. Evaluation of selected morphological traits and chemical compounds (expressed as mean value ± sd) in buckwheat seed in Austria in 2019–2021; Table S3. List of evaluated common buckwheat accessions; Table S4. Mass spectrometric data (negative ionization) for phenolic compounds detected in buckwheat (*Fagopyrum esculentum*) seeds by UHPLC-HRMS/MS analysis.

## Abbreviations

GAE—Gallic acid, API—Apigenin, CFA—Caffeic acid, CAE—Catechin, HYP—Hyperoside, CGA—Chlorogenic acid, ECAE—Epicatechin, IORI—Isoorientin, ORI—Orientin, IQ—Isoquercetin, VIT—Vitexin, IVIT—Isovitexin, PB—Procyanidin, RUT—Rutin, QCE—Quercetin, QCI—Quercitrin, NAR—Naringenin, TPC—Total polyphenols, TSW—1000-seed-weight, AA—Antioxidant activity, CP—Crude protein, RSC—Radical scavenging capacity, TE—Trolox equivalent, CZ—Czech Republic, AT—Austria.
